# Acute Severe Hyponatremia as a Serious Health Implication of Herbal Detox Regimens

**DOI:** 10.7759/cureus.3697

**Published:** 2018-12-06

**Authors:** Mohanad Soliman, William Fuller, Nida Usmani, Olalekan Akanbi

**Affiliations:** 1 Internal Medicine, University of Kentucky School of Medicine, Lexington, USA; 2 Internal Medicine, Presence Saint Joseph Hospital, Chicago, USA

**Keywords:** detox, hyponatremia, neurological, herbal medicine, herbs, beer-potomania

## Abstract

Hyponatremia is a serious health problem and can cause substantial morbidity and mortality as a result of osmotically induced cerebral edema if left untreated. Also, inappropriate rapid correction of chronic cases of hyponatremia can lead to osmotic demyelination with neurological impairment and death as consequences. It is defined as a serum sodium concentration less than 135 mmol/L.

Herbal detox regimens are gaining popularity with their easy access over the counter and not well studied adverse effects. We hereby present a case of a 67-year-old man who developed severe hyponatremia after starting a five-day kidney detox regime.

This regime consisted of drinking over a gallon (128 oz) of fluid daily, herbal tea with Uva Ursi leaves, juniper berries and several other ingredients. On the last day of this detox regime, he presented to the emergency department with a critical serum sodium level of 111 mmol/L associated with neurological symptoms. The purpose of this case report is to highlight the potential serious adverse effects associated with what is considered benign herbal medicine.

## Introduction

Over the past few years, the public interest in herbs as an alternative medical approach has grown and become more appealing. Although there is limited scientific evidence on the efficacy of some herbs (ginkgo, garlic, St. John’s wort, soy, and kava). Concerns over safety and unclear side effect profile render them from replacing standard medical therapies. Moreover, caution should be exercised consuming them especially when they are promoted as a way to cleanse toxins and promote health [1].

We hereby present a case of a 67-year-old man who started a strict five-day course of kidney detox regime. As a result he developed serious electrolytes imbalance that led him to be admitted to intensive care unit (ICU) for acute management and closer monitor.

## Case presentation

A 67-year-old male with no significant past medical history presented to the emergency department with tremors and lethargy for one day. He stated that he started a five-day course of kidney cleansing regimen and had followed the instructions to the letter. For five days, his oral intake was limited to drinking over a gallon (3.7 liters) of fluid daily along with herbal tea. On the fifth day, he started to experience vague weakness, fatigue, nausea, tremor and restlessness. On general examination, he looked anxious and restless. Vitals were blood pressure of 158/84 mmHg, respiratory rate of 28 cycles per minute, pulse of 88 beats per minute, temperature of 98 F, and oxygen saturation of 98% on room air. His cardiovascular and respiratory exams were unremarkable. Initial laboratory evaluation revealed a serum sodium of 111 mmol/L. Based on history and laboratory finding, a diagnosis of acute severe hyponatremia was made and the patient was admitted to the ICU. Treatment was started with intravenous (IV) fluid. Isotonic saline 0.9% was started initially at a rate of 125 cc/hour, after two hours serum sodium increased from 111 mmol/L to 112 mmol/L. So the decision was made to infuse hypertonic saline 3% at a rate of 45 cc/hour. That leads to forced diuresis and rapid rise of serum Na from 112 mmol/L to 120 mmol/L on two hours serum Na follow-up. In order to prevent this fast correction of serum Na dextrose 5% was started at a rate of 75 cc/hour and serum Na was monitored every four hours in intensive care setting. After 48 hours since the time of admission, the patient clinically improved, serum sodium increased to 129 mmol/L and intravenous fluid administration was discontinued. He was counselled to discontinue the detoxification regimen and follow-up with his primary care physician after discharge.

## Discussion

The term “herb” has been used loosely to refer not only to herbaceous plants but also to barks, stems, flowers, roots, and seeds. Under the current law, herbs are considered dietary supplements. So they are marketed without demonstrating safety and efficacy, as is required for pharmaceutical drugs [1].

Since prehistoric times, mankind has used herbs for medicinal and healing purposes [2]. While some herbs are safe in modest amounts, they can be toxic at higher concentrations. For example, whereas licorice root has been shown to be an effective therapy for treating duodenal and gastric ulcers, its excessive use can cause serious side effects such as high blood pressure, heart failure and even death [3].

Some herbal supplements have been advertised for health promotion as detoxification regimen. Inappropriate consumption of herbs along with imbalanced diet and lack of proper medical supervision may lead to grave outcomes. The detox regimen that we are presenting dictates a strict dietary plan of only drinking water and herbal tea for five consecutive days to allegedly cleanse the toxins out of the body. This imbalance between excessive free water and limited salt and solutes intake induces a state similar to beer potomania or tea and toast syndrome where reduction in dietary solute intake would largely limit the capacity of the kidney to excrete water. In order to produce 1 liter of maximally dilute urine, the kidneys require 50 mOsmol of solutes. This solute load is produced by urea generated from protein breakdown (10 g of protein produces around 50 mOsm of urea) [4]. Dietary sodium and potassium make up the remaining portion of the solute load. Based on a normal diet, daily intake of solute is between approximately 600 to 900 mOsm/d [4]. So in this patient, who was taking in a minimal amount of solute (daily average of 100 mOsm as a result of his low protein and minimal salt diet) and a renal diluting ability of 50 mOsmol per liter, any free water intake >2 L would exceed the diluting capacity of the kidney thereby placing the patient in a progressive hypotonic state.

Administration of Na containing intravenous fluid (solute load) would stimulate brisk diuresis causing rapid increase in serum Na levels, which may precipitate osmotic demyelination syndrome (ODS) in a chronically hyponatremic patient [4]. For that reason, the patient was admitted to the ICU for closer monitor of serum Na with a goal of correction limited to not more than 6-8 mEq of Na per 24 hours.

Our patient’s history of excessive water intake with lack of adequate solute consumption, clinical euvolemic state, and brisk diuresis in response to Na containing intravenous fluid made tea and toast syndrome the likely diagnosis.

After the initial correction of his hyponatremia during the first 48 hours, his serum Na remained in the range of 130 mEq/L to 134 mEq/L for the remainder of his hospitalization.

## Conclusions

Acute severe hyponatremia is a potentially life-threatening complication that developed in our patient. We hereby urge to increase the awareness amongst the public about the potentially serious metabolic and electrolyte derangements that can be associated with injudicious consumption of herbal regimens.
